# Molecular epidemiology and genetic dynamics of carbapenem-resistant hypervirulent *Klebsiella pneumoniae* in China

**DOI:** 10.3389/fcimb.2025.1529929

**Published:** 2025-02-14

**Authors:** Xiangchen Li, Sisi Chen, Yewei Lu, Weifeng Shen, Weixin Wang, Junli Gao, Junshun Gao, Pingyang Shao, Zhuxian Zhou

**Affiliations:** ^1^ College of Chemical and Biological Engineering, Zhejiang University, Hangzhou, Zhejiang, China; ^2^ Jiaxing Key Laboratory of Clinical Laboratory Diagnostics and Translational Research, Affiliated Hospital of Jiaxing University, Jiaxing, Zhejiang, China; ^3^ Cosmos Wisdom Mass Spectrometry Center of Zhejiang University Medical School, Hangzhou, Zhejiang, China; ^4^ Department of Clinical Laboratory, Affiliated Hospital of Jiaxing University, Jiaxing, Zhejiang, China

**Keywords:** *Klebsiella pneumoniae*, hypervirulence, carbapenem resistance, genomic epidemiology, plasmids

## Abstract

Carbapenem-resistant hypervirulent *Klebsiella pneumoniae* (CRhvKP) poses a significant global health threat due to its enhanced virulence and resistance. This study analyzed 5,036 publicly available *K. pneumoniae* genomes from China (2005–2023), identifying 1,538 CRhvKP genomes, accounting for 44.6% of carbapenem-resistant isolates and 69.5% of hypervirulent isolates. Predominant carbapenemases included *bla*_KPC_ (92.1%), with an increasing prevalence of *bla*_NDM_ and *bla*_OXA-48-like_ genes. Most isolates (93.6%) carried both aerobactin and yersiniabactin genes. The genetic background showed high diversity, characterized by 36 sequence types (STs) and 22 capsule types, with high-risk endemic STs such as ST11, ST15, and ST23 being predominant. ST23 demonstrated enhanced virulence, whereas ST11 carried more resistance genes but showed minimal presence of *iroBCDN* genes. A core genome MLST analysis revealed that 89.0% of CRhvKP isolates clustered into 131 clonal groups, indicating widespread dissemination, particularly in eastern China. CR and hv plasmids, primarily IncF, IncH, and IncR types, showed distinct community structures, with CR plasmids demonstrating higher mobility and diversity. Crucially, we identified 40 CR-hv convergent plasmids across five STs, likely resulting from plasmid fusions, which have become increasingly prevalent in eastern China over the last decade. Furthermore, chromosomal integration of hv genes and *bla*_KPC-2_ was detected, underscoring the stable inheritance of these traits. Class 1 Integrons were present in 84.5% of CRhvKP strains, most notably in ST11 and least in ST23. These integrons harbored genes that confer resistance to various antibiotics, including *bla*_IMP_ and *bla*_VIM_, with their content varying across different STs. This study highlights the genetic complexity, rapid dissemination, and increasing prevalence of CRhvKP in China, emphasizing the urgent need for enhanced genomic surveillance and targeted interventions to mitigate the threat posed by these multidrug-resistant and hypervirulent strains.

## Introduction

*Klebsiella pneumoniae* (KP) is responsible for a wide range of infections, including pneumonia, urinary tract infections, bloodstream infections and liver abscesses, and continues to be a prevalent nosocomial pathogen (Bengoechea and Sa Pessoa, 2019). According to the 2023 China Antimicrobial Surveillance Network (CHINET), KP is now the second most frequently isolated bacterium in clinical settings in China, after *Escherichia coli*, with KP’s resistance to meropenem rising steadily from 2.9% in 2005 to 30.0% in 2023 (Pan et al., 2024).

KP is a microorganism capable of readily acquiring genes encoding hydrolyzing enzymes, often hosted on mobile genetic elements like plasmids and integrons, thereby accelerating their spread. Over the past two decades, KP has evolved into two different evolutionary genetic lines: classical KP (cKP) and hypervirulent KP (hvKP) (Mendes et al., 2023). cKP commonly carries several determinants that confer multidrug resistance (MDR), especially carbapenem resistance (CR) related carbapenemase genes (*bla*Carbs), such as *bla*_KPC-2_ (class A), *bla*_NDM_ (class B), *bla*_IMP_ (class B), *bla*_VIM_ (class B), *bla*_OXA-48-like_ (class D), and causes infections in hospitalized or otherwise immunocompromised patients (Hu et al., 2024). Besides, MDR-cKP strains frequently produce extended-spectrum beta-lactamases (ESBLs), including CTX-M-15 and SHV-12, which confer resistance to third-generation cephalosporins (Riwu et al., 2020). hvKP poses a significant threat as it can cause severe infections in otherwise healthy individuals with high mortality rates (Das, 2024). hvKP isolates can generate an enhanced level of capsule, which can be detected by a string test, and a positive result is iconic as a typical hypermucoviscous phenotype (Choby et al., 2020). Recently, the molecular definition of hvKP strains has been used more broadly (Chen et al., 2024). In certain studies, isolates that possess both the *iucA* and *rmpA*/*A2* genes are categorized as hvKP (Spadar et al., 2023; Hu et al., 2024).

Traditionally, CR or MDR and hv are associated with distinct KP populations, with CR in cKP strains and hv rarely linked to resistance (Mendes et al., 2023). However, since both traits can be horizontally transferred, they can merge within the same strain, blurring lines between CR and hypervirulent strains. The evolution of hv or CR plasmids has led to the emergence of carbapenem-resistant hypervirulent KP (CRhvKP) (Gu et al., 2018). Tian et al. previously proposed three hypotheses for the formation of CRhvKP, which include hvKP acquiring CR plasmids, carbapenem-resistant *K. pneumoniae* (CRKP) acquiring hv plasmids, and the convergence of virulence and carbapenem resistance in a single plasmid, carried by either hvKP or CRKP strains (Tian et al., 2022). Recently, CRhvKP has been causing severe infections and hospital outbreaks in China (Gu et al., 2018; Xie et al., 2021; Yang et al., 2022). Subsequently, a growing number of researchers worldwide have reported the emergence of CRhvKP strains (Wu et al., 2022; Kochan et al., 2023; Nguyen et al., 2024). With limited treatment options and the potential for subsequent metastatic spread, the outcome could be devastating.

To better understand the molecular epidemiology and genetic landscape of CRhvKP in China, this study collected all available CRhvKP genomes from China in the Bacterial and Viral Bioinformatics Resource Center (BV-BRC) database. We performed a comprehensive genomic analysis of genetic diversity, antimicrobial resistance and virulence, phylogenetic relationship, clonal transmission and plasmid profile of CRhvKP in the country.

## Materials and methods

### Genome collection and quality control

We retrieved all public KP genome assemblies from BV-BRC database as of September 4, 2024 (Olson et al., 2023). The search criteria used were: “GENOMES = *Klebsiella pneumoniae*”, “isolation country = China”, “genome status = WGS”, “genome quality = good” and “host group = human”. The corresponding metadata for these genomes was also obtained from the BV-BRC database and cross-verified manually using the NCBI GenBank database. Quality assessments for downloaded genomes were calculated with QUAST v5.2.0 and fastANI v1.33, respectively (Gurevich et al., 2013; Musiał et al., 2024). Genomes with over 95% average nucleotide identity and at least 80% coverage relative to the reference genome HS11286 (NCBI RefSeq: NC_016845) were selected for further analysis.

### Genotyping and classification

Kleborate v3.0.8 identified MLST profiles, virulence loci such as *ybt*, *clb*, *iro*, *iuc*, and *rmpA/A2*, predicted capsule types (serotype), and detected acquired antimicrobial resistance (AMR) genes, including *bla*ESBLs and *bla*Carbs (Lam et al., 2021). We defined isolates carrying at least one of *bla*Carbs as CRKP. Isolates possessing both the *iucA* and *rmpA*/*A2* genes were categorized as hvKP (Hu et al., 2024). Isolates that met both criteria were defined as CRhvKP (Chen et al., 2024).

### Phylogenetic and population structure analysis

The core genome alignment and SNP calling (cgSNP) were conducted using Parsnp v1.2 from the HarvestTools suite (Treangen et al., 2014). A maximum likelihood phylogenetic tree based on the concatenated alignment was inferred using FastTree v2.1 with the GTR+GAMMA model (Price et al., 2010). The resulting phylogenetic tree with metadata was visualized using the interactive Tree of Life (iTOL) web application (Letunic and Bork, 2021).

For cgMLST allele calling, the chewBBACA v3.3.9 was employed with a public 2,358-gene typing scheme derived from 14,254 genomes of *K. pneumoniae*, *K. variicola*, and *K. quasipneumoniae*, as provided by the RIDOM Nomenclature Server (https://www.cgmlst.org) (Silva et al., 2018). Pairwise cgMLST distances between strains were calculated using cgmlst-dists v0.4.0 (https://github.com/tseemann/cgmlst-dists), based on the core genes present in more than 95% of the collected genomes. A minimum spanning tree (MST) was constructed using GrapeTree v1.5.0 with the MSTv2 algorithm (Zhou et al., 2018). The cgMLST-based genomic clustering network was visualized and analyzed with Cytoscape v3.10.2 (Shannon et al., 2003).

### Plasmid sequence reconstruction and typing

Plasmid sequences were identified and reconstructed from the genome assemblies with MOB-Suite v3.19 (Robertson and Nash, 2018). This process involved using MOB-typer for relaxase and replicon typing, generating MOB-cluster codes, and determining host range. Plasmid mobility was predicted based on relaxase (MOB), mating pair formation (MPF) complex, and *oriT* gene annotations. In summary, plasmids were classified as conjugative if they had both relaxase and MPF, mobilizable if they contained either relaxase or *oriT* but lacked MPF, and non-mobilizable if they had neither relaxase nor *oriT* (Smillie et al., 2010).

### Plasmid similarity estimation

Pairwise distances between plasmids were calculated using Mash v2.2, which estimates sequence similarity by converting sequences into fixed-length MinHash sketches (Ondov et al., 2016). The Mash distance (ranging from 0 for nearly identical sequences to 1 for completely dissimilar sequences) was used to assess similarity (1-Mash distance), with a k-mer length of 13 and a sketch size of 5,000.

For plasmid community detection, the Louvain algorithm was employed to identify clusters by optimizing modularity through iterative expectation-maximization, implemented via the Python module python-Louvain v0.16 (Blondel et al., 2008). We calculated the Louvain partition for the network and selected nodes from communities containing at least 10 members.

### Plasmid annotation and pangenome analysis

Plasmids were annotated using Prokka v1.13.4 (Seemann, 2014). Insertion sequences (ISs) were detected by digIS v1.2 (Puterová and Martínek, 2021). Pangenome analysis was performed by Panaroo v1.5.0 (Tonkin-Hill et al., 2020). Abricate v1.0.1 (https://github.com/tseemann/abricate) was used to identify acquired AMR genes from the CARD database and virulence factors (VFs) from the VFDB database, using a threshold of 90% sequence identity and 90% coverage (Liu et al., 2022; Alcock et al., 2023). Circular alignment of the plasmids was performed and visualized with BRIG v0.95 (Alikhan et al., 2011).

### Integron detection

Annotations for integron integrases, *attC*/*attI* sites, and integron promoters (P_c_/P_int_) were generated using IntegronFinder v2.0.5 with default parameters, except for the options “–local-max” (Néron et al., 2022). IntegronFinder classifies integron elements into three types: (1) Complete integrons, which include an integrase and at least one *attC* site; (2) In0 elements, consisting of an integrase without any nearby *attC* site; and (3) CALIN elements, clusters of *attC* sites that lack associated integron integrases, comprising at least two *attC* sites. The AMR genes located on the integron-associated gene cassettes were also detected by Abricate v1.0.1 based on the CARD database.

## Results

### Overview of CRhvKP isolates among the public KP genomes from China

**Figure 1:**
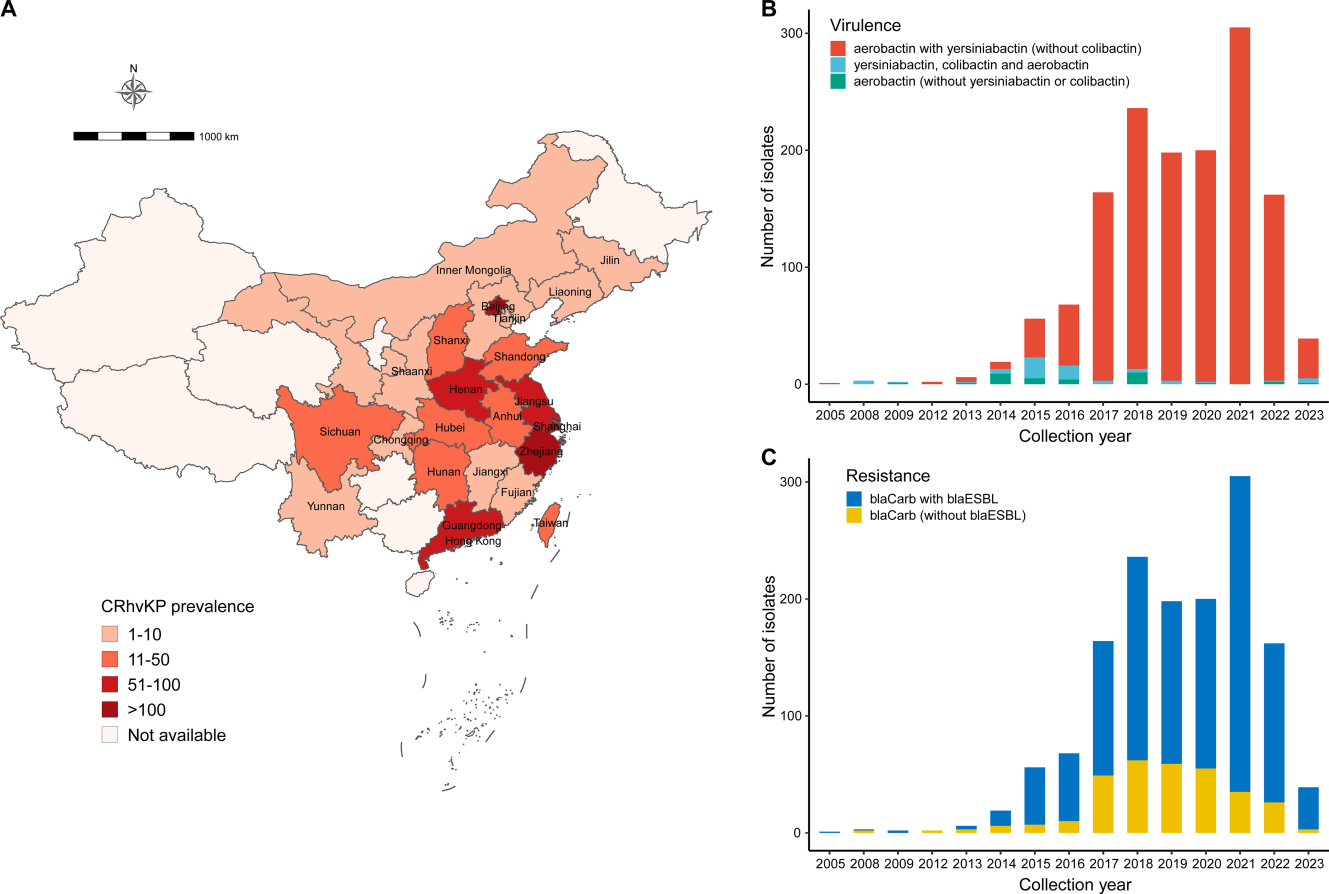
Prevalence of CRhvKP in China from 2005 to 2023. **(A)** Geographic distribution of the 1,538 CRhvKP isolates. The gradient red backgrounds represent the number of isolates. **(B, C)** show the distribution of the number of isolates by sampling year based on virulence classes and whether they simultaneously encode the *bla*ESBLs, respectively.

We collected 5,036 KP genomes with metadata from China after screening a database on September 4, 2024. Genotyping with Kleborate identified 3,449 isolates as CRKP and 2,212 as hvKP, with 1,538 classified as CRhvKP (44.6% of CRKP and 69.5% of hvKP; **Supplementary Table S1**). These CRhvKP isolates were reported from 26 province-level divisions, primarily concentrated in the central and eastern regions (**Figure 1A**). The top five regions by isolate count were Zhejiang (n=629), Shanghai (n=288), Beijing (n=127), Jiangsu (n=77), and Henan (n=63). The earliest sequenced CRhvKP in China was isolated from Zhejiang in 2005. Over the past 20 years, CRhvKP isolates have significantly increased, peaking in 2021 before declining.

According to the virulence detection of Kleborate, 1,439 (93.6%) isolates had a virulence score of 4, indicating the presence of both aerobactin and yersiniabactin (**Figure 1B**). Additionally, 63 strains (4.0%) had the highest score of 5, carrying aerobactin, colibactin, and yersiniabactin, while only 36 strains (2.3%) scored 3, indicating the presence of aerobactin alone. Notably, since 2019, CRhvKP strains carrying only aerobactin have become increasingly rare.

*bla*_KPC_ were detected in 1,224 (92.1%) of collected CRhvKP isolates and were the most prominent *bla*Carb. *bla*_OXA-48-like_ was the second most frequent (n=273, 17.8%), followed by *bla*_NDM_ (n=53, 3.4%), *bla*_IMP_ (n=26, 1.7%) and *bla*_VIM_ (n=1, 0.1%). 39 strains carried multiple *bla*Carbs: 34 encoded *bla*_KPC_ and *bla*_NDM_, 2 had *bla*_KPC_ and *bla*_OXA-48-like_, 2 had *bla*_NDM_ and *bla*_OXA-48-like_, and 1 had *bla*_KPC_ and bla_IMP_. There has been a significant increase in the proportion of strains encoding *bla*_OXA-48-like_ since 2021 (**Supplementary Figure S1**). Furthermore, 96.9% (n=1,491) of collected CRhvKP isolates contained resistance determinants for 3 or more classes of antibiotics, and the majority (n = 1,212, 78.8%) contained *bla*ESBLs. Over the past five years, the proportion of CRhvKP strains co-harboring both *bla*Carbs and *bla*ESBLs has risen annually, from 70.1% in 2017 to 92.3% in 2023 (**Figure 1C**).

### Phylogenetic structure of CRhvKP isolates

**Figure 2:**
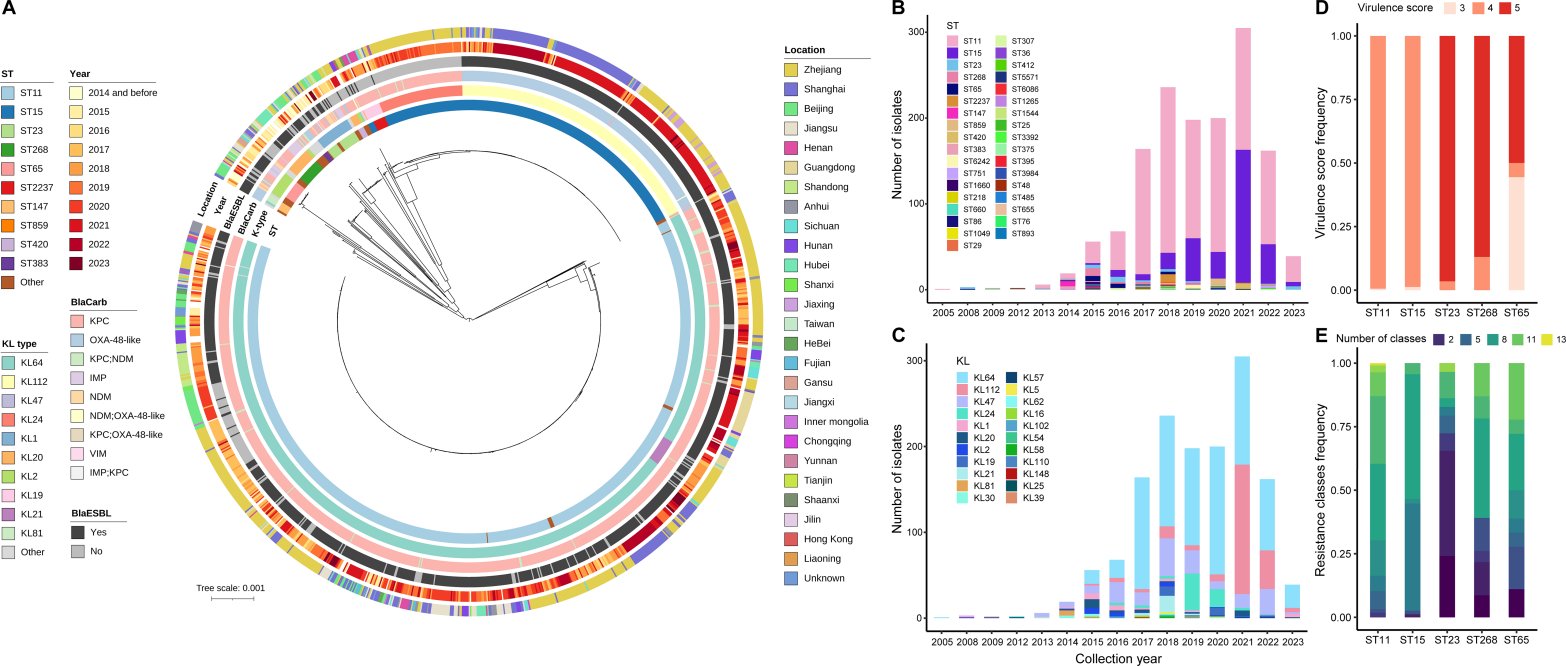
Genetic features of the 1,538 CRhvKP isolates from China. **(A)** The cgSNP-based phylogenetic tree was visualized using the iTOL and mid-point rooted. The concentric circles, from inner to outer, represent ST, KL, *bla*Carb class, presence of *bla*ESBL, collection year and location. The scale bar indicates the number of nucleotide substitutions per site. **(B, C)** display the frequency distribution of different STs and KL-types by sampling year, respectively. **(D, E)** illustrate the frequency distribution of virulence scores and the number of acquired AMR classes among the five predominant STs, respectively.

To gain molecular insights, we reconstructed a phylogenetic tree for all 1,538 CRhvKP isolates, mapping it with ST, KL type, *bla*Carb class, *bla*ESBL presence, collection year, and location (**Figure 2A**, **Supplementary Table S1**). The analysis showed that strains clustered by STs and KL types, identifying 36 STs, with the top five being ST11 (68.7%), ST15 (21.1%), ST23 (1.9%), ST268 (1.5%), and ST65 (1.2%). Although ST11 was the most prevalent, it was genetically distant from ST15, ST23, and ST268. The proportion of ST15 increased over the past five years, reaching 50.8% in 2021 (**Figure 2B**). We identified 134 capsular types (KLs), with the top five being KL64 (54.4%), KL112 (15.6%), KL47 (13.3%), KL24 (5.1%), and KL1 (2.1%) (**Figure 2C**).

Notably, ST11 and ST15 had a virulence score of 4 (>99%), while ST23, ST268 and ST65 had a score of 5 in 96.6%, 87% and 50% of cases, respectively (**Figure 2D**). Besides, 100% of ST65, 93.1% of ST23 and 56.5% of ST268 strains encoded the *iroBCDN* virulence gene cluster. In contrast, only 6.2% of ST11 and 0.3% of ST15 strains carried these genes (**Supplementary Figure S2**). Moreover, the number of acquired AMR classes varied significantly among the major STs (**Figure 2E**). The proportion of strains carrying more than six antibiotic resistance classes was highest in ST11 at 83.6%, followed by ST65 (61.1%), ST268 (60.9%), and ST15 (55.2%), while the proportion in ST23 was only 17.2%.

### Molecular epidemiology of CRhvKP in China

**Figure 3:**
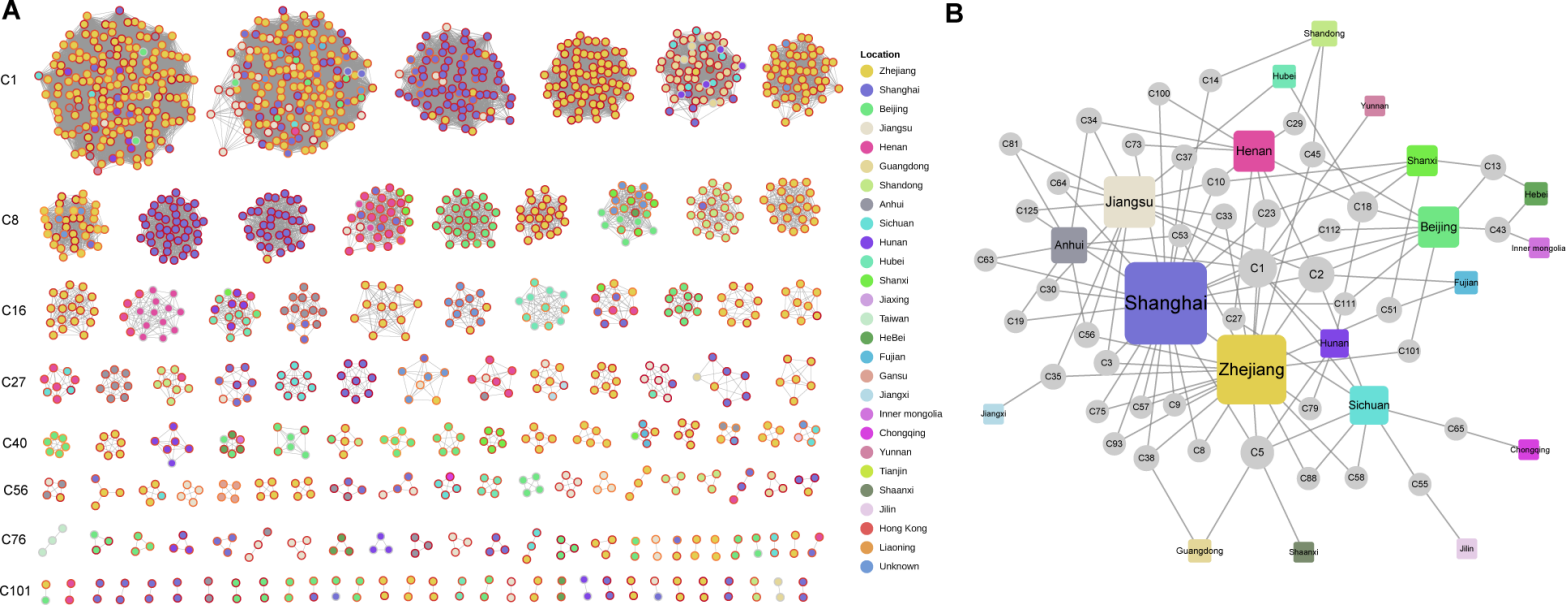
Clonal clustering profile of collected CRhvKP isolates. **(A)** Cytoscape view of the network for the 131 clonal clusters. Each node represents a strain, and edges signify allele differences of fewer than 15. The internal color of the nodes represents the sampling regions, and the node rings range from yellow to dark red, indicating sampling times from before 2014 to 2023. **(B)** A bipartite network showing the relationship between 42 clusters across multiple province-level divisions. Circular and square nodes represent clusters and sampling sites, respectively. The size of each node represents its degree, indicating the number of connections it has to other nodes.

Using a threshold of 15 cgMLST allele differences (Lan et al., 2020), 1,369 isolates (89.0%) were grouped into 131 clonal clusters, ranging in size from 2 to 180 members, and ordered from largest to smallest, from C1 to C131 (**Figure 3A**, **Supplementary Table S2**). Compared to the clustering rates of ST11 (91.2%) and ST15 (95.7%), ST23 had a lower clustering rate of 41.4%. Clustered samples of over 10 strains were detected in 9 province-level divisions, ranked by clustering rate: Guangdong (94.1%, 48/51), Zhejiang (92.2%, 599/650), Henan (90.5%, 57/63), Beijing (88.2%, 112/127), Anhui (88.1%, 37/42), Shanghai (87.8%, 253/288), Jiangsu (87.0%, 67/77), Sichuan (86.1%, 31/36) and Shandong (83.7%, 36/43), suggesting the severe CRhvKP epidemic in these areas.

Strains from 42 clusters were sampled from 20 province-level divisions, with two major clusters, C1 and C2, each containing over 100 members. C1 included 180 strains collected from eastern (Shanghai, Zhejiang, Jiangsu), northern (Beijing), central (Anhui, Hunan), and western (Sichuan, Yunnan) regions over 18 years (2005–2023). C2 comprised 164 strains from eastern (Shanghai, Zhejiang, Jiangsu, Fujian), northern (Beijing), central (Henan), and western (Sichuan) regions, collected over 7 years (2015–2022). A bipartite network analysis indicated that eastern China, especially Shanghai, Zhejiang, and Jiangsu, served as a transmission hub for CRhvKP, linking strains from 13 regions, excluding Hebei, Inner Mongolia, Chongqing and Jilin (**Figure 3B**). Notable connections also emerged from Beijing (linked to Hebei and Inner Mongolia) and Sichuan (linked to Chongqing).

### Plasmid profiling and incompatibility grouping of CRhvKP

A total of 8,645 plasmids were detected based on MOB-Suite, with the number of plasmids per strain ranging from 1 to 11. The number of plasmids in ST15 strains was significantly higher than in the other four main STs (Kruskal–Wallis test P < 0.01, **Supplementary Figure S2**). Plasmids ranged in length from 1.2 to 365.0 kb. Twelve types of replicon families were detected from these plasmids, with the IncF being the most prevalent replicon (41.3%, 3569/8645).

**Figure 4:**
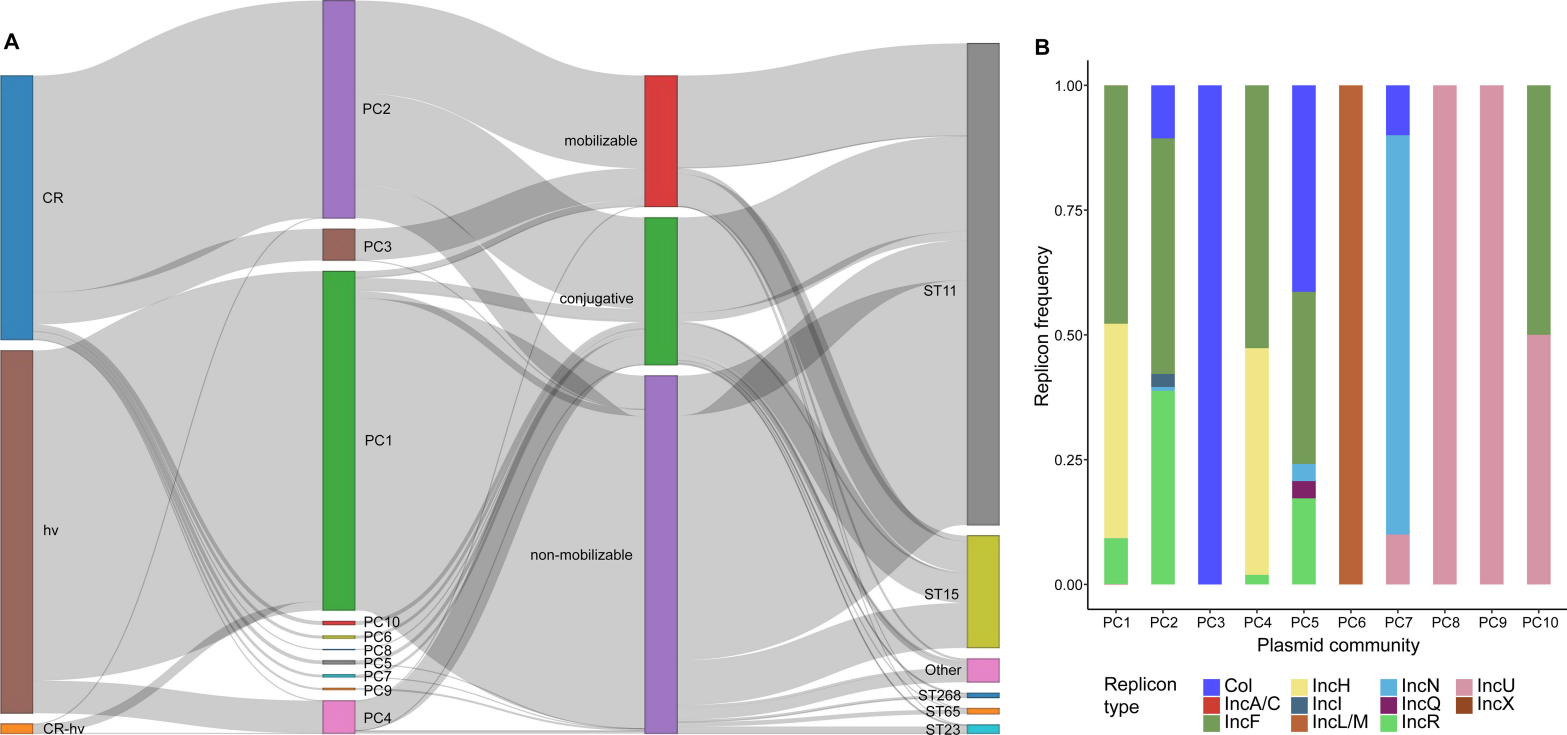
Genetic profiling of CR and hv plasmids in CRhvKP from China. **(A)** Sankey diagram illustrating the connections between CR and hv plasmids and their phenotypes, mobilities and PCs, and strains’ STs. The thickness of each line is proportional to the number of isolates. **(B)** The frequency distribution of the replicon types detected in different PCs of CR and hv plasmids.

A total of 1,496 hv plasmids carrying hv genes were identified in 1,494 strains, and 1,546 CR plasmids encoding *bla*Carbs were found in 1,519 strains (**Supplementary Table S3**). Additionally, 42 strains carried both hv and *bla*Carb genes on a single plasmid. Using the Louvain algorithm with a Mash distance threshold of 0.95 (Matlock et al., 2021), we identified 10 plasmid communities (PC1 to PC10), with cluster sizes ranging from 15 to 1,260 (**Figure 4**). PC1 (n=1,254), PC4 (n=227) and PC5 (n=126) were broadly distributed among ST11, ST15, ST23 and other STs, whereas PC2 was predominantly found in ST11 (97.7%, 979/1002), and PC3 was primarily associated with ST15 (96.4%, 240/249). Plasmid mobility analysis showed that most PCs were conjugative or mobilizable, except for PC1 and PC9, which were largely non-mobilizable.

CR and hv plasmids were assigned to different PCs, with CR plasmids showing greater diversity across 9 PCs, where 64.8% (1002/1546) clustered in PC2. Conversely, 83.8% (1254/1496) of hv plasmids were predominantly in PC1, with an additional 15.1% (226/1496) in PC4. Ten different replicons were identified in both CR and hv plasmids, with IncF (74.5%, 2236/3002), IncH (43.5%, 1306/3002), IncR (31.0%, 930/3002), and Col-like (14.9%, 447/3002) being the most common. Specifically, PC1 and PC4 predominantly featured IncF/IncH, PC2 was mainly composed of IncF/IncR, and PC5 included a mix of Col-like/IncF/IncR. PC10 was characterized by IncF/IncU, while PC3, PC6, PC7, PC8 and PC9 were primarily represented by Col-like, IncL/M, IncN, IncU and IncU, respectively (**Supplementary Figure S3**).

### Fusion plasmids carrying both *bla*Carb and hv genes

A total of 40 CR-hv convergent plasmids were identified, carrying both *bla*Carb (38 *bla*_KPC-2_, 2 *bla*_NDM-1_) and hv genes: 32 were classified as PC1, six as PC4, one as PC2, and one as other (**Supplementary Table S3**). PC1 was found in ST11, ST23, ST268, ST660 and ST1660, comprising hybrid plasmids from seven replicon types (IncF, IncH, IncR, IncA/C, IncN, IncU and IncX). In contrast, PC2 and PC4 were only detected in ST11 and were solely IncF/IncR hybrids. Since their first identification in Zhejiang isolates in 2013, CR-hv convergent plasmids have been consistently detected through 2022, primarily in eastern China (85.0%, n=34). Strains carrying these plasmids belong to 13 clonal clusters, with C2 being the largest at 37.5% (n=15), indicating a crucial role of these plasmids in spreading both CR and hv traits.

**Figure 5:**
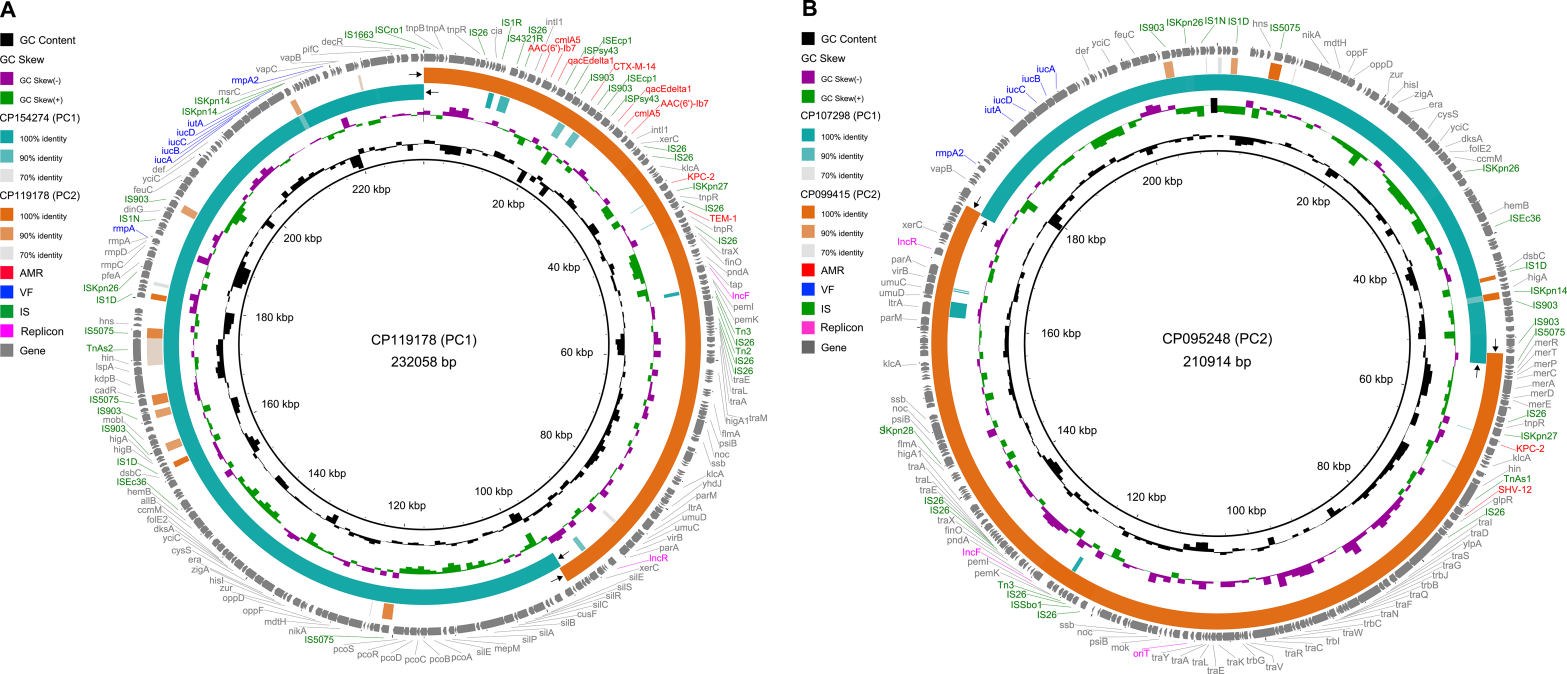
Genetic map of CR-hv convergent plasmids of PC1 and PC2. **(A)** Alignment of the CR-hv convergent plasmid (CP119178) from PC1 with the hv plasmid (CP154274) from PC1 and the CR plasmid (CP119178) from PC2. **(B)** Alignment of the CR-hv convergent plasmid (CP095248) from PC2 with the hv plasmid (CP107298) from PC1 and the CR plasmid (CP099415) from PC2. The black arrows indicate regions of homologous recombination between CR and hv plasmids. The outer ring shows all protein-coding genes, color-coded: AMR in red, VF in blue, IS in green, and replicons in magenta, with others in gray.

To investigate the genetic features of CR-hv convergent plasmids, we selected two complete circular IncF/IncR plasmid sequences from PC1 and PC2 as references: CP119178 (226.6 kb) and CP095248 (206.0 kb). Sequence alignments revealed that both plasmids resulted from fusion events in small homologous regions. CP119178, a non-mobilizable plasmid, formed by fusing a 133.3 kb sequence from the IncF/IncH plasmid CP154274 (PC1) with a 93.6 kb sequence from the IncF/IncR plasmid CP119178 (PC2), linked by a 328 bp region encoding a hypothetical protein (**Figure 5A**). In contrast, CP095248, a conjugative plasmid, merged a 118.3 kb sequence from CP107298 (PC1) with an 88.0 kb sequence from CP099415 (PC2), connected by a 1,345 bp IS*5075* (IS*110*-like) transposon and a 255 bp region encoding a hypothetical protein (**Figure 5B**). Additionally, the replicons (IncF and IncR) in both CP154274 and CP119178 were located on segments derived from the fused PC2 plasmid.

### Chromosomal integration of *bla*Carb and hv genes from plasmids

We identified hv and *bla*Carb genes on the chromosomes of several strains in addition to plasmids. A total of 28 strains carried hv genes (*iucABCD*, *iutA* and *rmpA2*) on their chromosomes, with 41 having them exclusively located there. Among these, 21 were ST11, 6 were ST2237, and one belonged to ST1544. Notably, a ST11 strain named XHKPN391, isolated from Shanghai in 2018, carried both *bla*_KPC-2_ and hv genes on its chromosome (CP066915), with hv genes found only there. In the clonal cluster (C10) containing XHKPN391, none of the other 30 members had *bla*_KPC-2_, although 15 strains carried hv genes.

**Figure 6:**
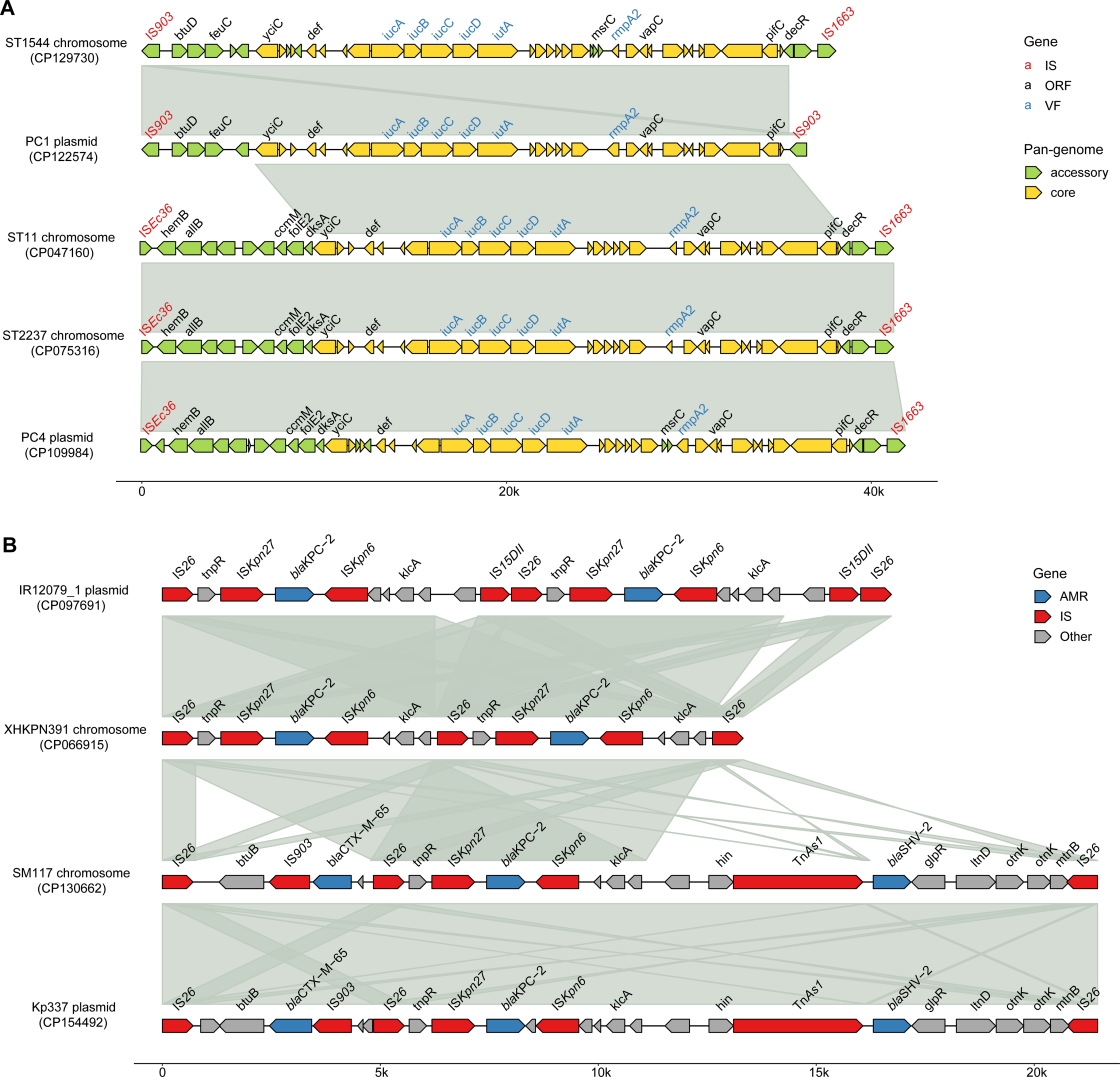
Schematic representation of the CR and hv genetic environments on CRhvKP chromosomes and plasmids. **(A)** The multiple sequence alignments of chromosomal hv gene regions in ST11, ST1544 and ST2237 with those on plasmids in PC1 and PC4. **(B)** The multiple sequence alignments of the *bla*_KPC-2_ gene environments on the chromosomes of ST11 strains XHKPN391 and SM117 with those on plasmids in ST11 strains IR12079_1 and Kp337. Arrows indicate genes and the known gene names are labeled above the arrows. Shaded areas highlight regions of homology (>99% nucleotide identity). The image was generated using the R package gggenomes (https://github.com/thackl/gggenomes).

In ST11 and ST2237, the hv genes are situated within a 40.8 kb region, bordered by IS*Ec36* (IS*3*-like) and IS*1663* (IS*110*-like) (**Figure 6A**). In contrast, ST1544 features hv genes within a 35.6 kb segment, flanked by two IS*903* transposons. BLAST results showed that the two chromosomal hv regions on ST11 and ST2237 closely matched the PC1 plasmid (99.9% identity, 99% coverage), while the chromosomal hv region on ST1544 was highly homologous to the PC4 plasmid (99.9% identity, 95% coverage), featuring IS*903* upstream and IS*1663* downstream. Additionally, comparative analysis revealed that these hv regions shared 30 core genes, which make up 52-83% of each region.

Two ST11 strains, XHKPN391 and SM117, carried *bla*Carbs on both their chromosomes and plasmids. XHKPN391 harbored two *bla*_KPC-2_ genes on its chromosome (CP066915) and one on a plasmid, while SM117 contained one *bla*_KPC-2_ on its chromosome (CP130662) and both a *bla*_KPC-2_ and *bla*_NDM-5_ on two plasmids. The *bla*_KPC-2_ in both strains were located within genetic regions flanked by two IS*26* (IS*6*-like) transposons, measuring 13 kb and 21 kb, respectively (**Figure 6B**). A BLAST analysis showed that the IS*26*-flanked *bla*_KPC-2_ regions in XHKPN391 and SM117 shared high synteny and identity (>99%) with plasmids from ST11 isolates IR12079_1 (CP097691) and Kp337 (CP154492), respectively. The core genetic structure for *bla*_KPC-2_ in these replicons was characterized by IS*26*, IS*Kpn6*, *bla*_KPC-2_, IS*Kpn27*, *tnpR*, and IS*26*.

### Integron profiling of CRhvKP isolates

**Figure 7:**
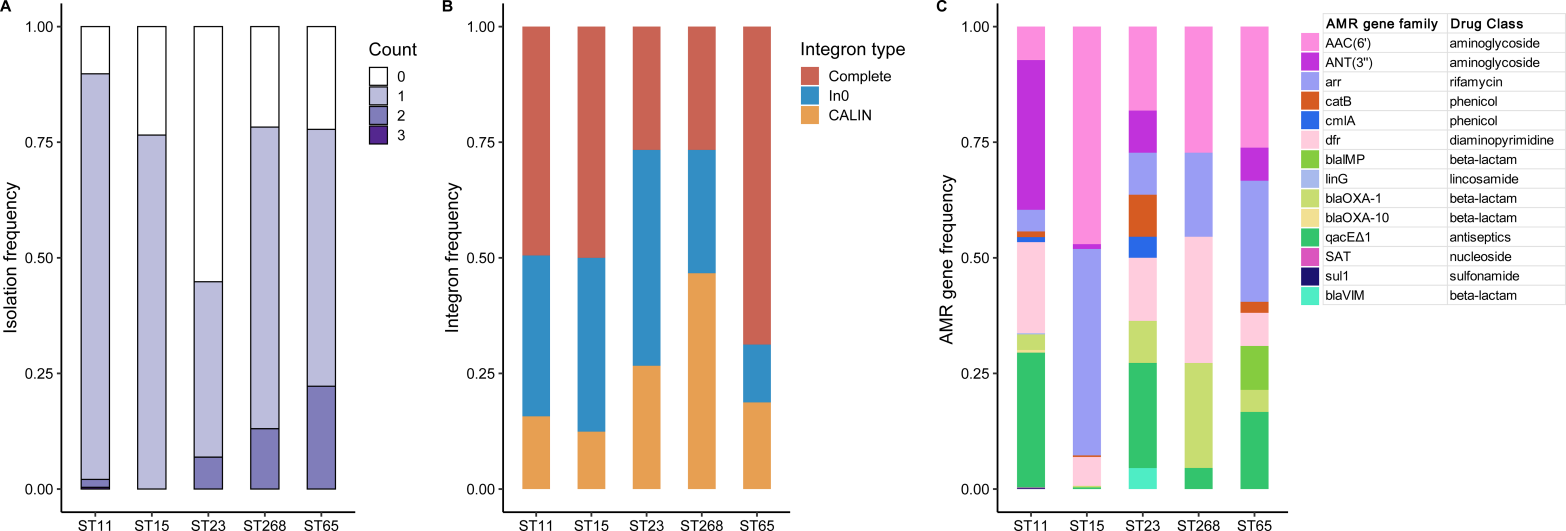
Integron profiling of the five major STs. **(A)** The frequency distribution of the number of integrons detected in each strain of the five major STs. **(B)** The proportional distribution of different types of integrons detected in the five major STs. **(C)** The frequency distribution of AMR gene families in the integron-related gene cassettes in the five major STs.

A total of 1,336 class 1 integrons were identified across 1,300 (84.5%) CRhvKP isolates, including 725 complete integrons, 448 In0, and 163 CALIN elements. Integrons were found in more than 75% of ST11, ST15, ST268, and ST65 isolates, while the detection rate in ST23 strains was significantly lower, at 44.8% (**Figure 7A**). The predominant integron type in ST11, ST15 and ST65 strains was the complete integron, while ST23 and ST268 were primarily characterized by In0 and CALIN elements, respectively (**Figure 7B**). We identified 14 AMR gene families from the mobile gene cassettes within these integrons, conferring resistance to various antibiotics and antiseptics (**Figure 7C**). Notably, four integrons in ST65 carried *bla*_IMP_, and one in ST23 contained *bla*_VIM_, underscoring their role in *bla*Carb dissemination. Furthermore, the integron-related AMR gene profiles varied significantly across different STs. For example, ST11 mainly carried *ANT(3’’)*, *dfr* and *qacEΔ1*, while ST15 primarily harbored *AAC(6’)* and *arr*.

## Discussion

In this study, we investigated molecular epidemiology and genetic architecture of CRhvKP in China, shedding light on these strains’ genetic diversity and epidemiological spread. While the initial report of CRhvKP in China was in 2015 (Zhang et al., 2015), our genomic data reveal the earliest CRhvKP strain dating back to 2005, and over the nearly two decades since then, the numbers of CRhvKP in China have increased significantly. The proportion of CRhvKP among CRKP isolates circulating in China is higher compared to other nations (Wu et al., 2022). The predominance of carbapenemase genes, particularly *bla*_KPC_ (92.1%), aligns with previous studies indicating the widespread presence of this enzyme in China. Worryingly, the rising detection of alternative carbapenemases like *bla*_OXA-48-like_ and *bla*_NDM_, along with *bla*ESBLs, aggravates MDR issues, underscoring an urgent need for effective treatment options. This diversity in AMR genes indicates the evolutionary pressures exerted by extensive antibiotic use, emphasizing the need for judicious antimicrobial stewardship.

Our data reveal a complex diversity of STs and KLs within the CRhvKP population in China, which complicates the management and treatment of infections caused by these strains. High-risk endemic STs, such as ST11, ST15 and ST23, were predominant, exhibiting distinct virulence and resistance profiles. ST11 and ST15 were international high-risk CRKP clones reported mainly from Asia and Europe and responsible for nosocomial transmission and various care center outbreaks (Feng et al., 2023; Rodrigues et al., 2023; Shi et al., 2024). It is noteworthy that ST11 is detected more often, possibly due to heightened scrutiny and screening for CRKP, while hvKP lineages like ST23 might be under-detected because of their relatively lower MDR rates (Arcari and Carattoli, 2023). Although serotypes KL1 and KL2 are usually linked to hvKP, the emergence of ST11 and ST15 CRKP strains with hv plasmids has highlighted KL64 and KL112 in the context of CRhvKP. While these serotypes were previously associated with cKP in hospital settings, their specific contributions to virulence are still unclear (Zhou et al., 2023). Furthermore, we found that both ST11 and ST15 predominantly lack the *iroBCDN* virulence gene cluster, consistent with Jia et al.’s findings that ST11-KL64 CRKP strains improve fitness by eliminating this cluster, thereby highlighting the genetic basis for the dominance of the hv-CRKP clone in China (Jia et al., 2024). These findings underscore the need for future studies to explore the dynamic interplay between resistance and virulence traits in CRhvKP, which could provide critical insights into their evolution and inform more effective prevention and treatment strategies.

High-resolution cgMLST analysis indicated that most of isolates likely resulted from clonal transmission, signifying that CRhvKP has become a major player in CRKP infections in China. We identified evidence of CRhvKP transmission across 13 provinces and municipalities, with a notable hub in eastern China. Major clusters from 2005 to 2023 included strains from various regions, reflecting ongoing spread. Eastern China, with its economic vibrancy and abundant medical resources attracting many migrant workers, is likely the reason for the concentration of sequenced strains, underscoring its role in the dissemination of CRhvKP. Recently, Hu et al. conducted a phylogenetic and spatiotemporal analysis of CRKP and revealed that Shanghai, the largest city in eastern China, serves as the major transmission hub, influencing the geographical prevalence of KL47 and KL64 strains across China (Hu et al., 2024). This widespread dissemination underscores the role of regional hubs in the spread of CRhvKP and highlights the need for enhanced surveillance and intervention strategies in these areas.

Plasmid profiling revealed that *bla*Carb and hv genes in CRhvKP were predominantly plasmid-borne, with IncF, IncH and IncR being the common replicon types. The hv plasmids, limited to two communities (PC1 and PC4) across diverse STs, possess a genetic structure analogous to the renowned virulence plasmids pLVPK (CG43, ST86/K2) and pK2044 (NTUH-K2044, ST23/K1), respectively (Chen et al., 2004; Wu et al., 2009). These hv plasmids were typically non-mobilizable due to the lack of partial conjugative elements such as a complete T4SS gene cluster. A recent study by Xu et al. demonstrated that, under specific conditions, a non-conjugative virulence plasmid can be mobilized by a conjugative IncF plasmid from hvKP strain into ST11 CRKP strains (Xu et al., 2021). In contrast to hv plasmids, CR plasmids exhibit high diversity and are largely conjugative. It appears that hvKP strains find it easier to acquire conjugative CR plasmids than for CRKP strains to acquire non-conjugative virulence plasmids (Yong et al., 2022).

Plasmid fusion has been reported in several studies, with the majority of these known fusion processes being mediated by IS elements through homologous recombination (Xie et al., 2018; Chen et al., 2019). The first reported hybrid resistance and virulence plasmid was obtained from a clinical hvKP strain collected in 2013 in China (Zhang et al., 2016). Recently, Xie et al. reported a conjugative hybrid plasmid (IncF/IncH) was formed by fusion of a non-conjugative pLVPK-like plasmid and a conjugative *bla*_KPC-2_-bearing plasmid (Xie et al., 2021). In this study, we also identified 40 hybrid plasmids in five STs that have combined CR and hv traits through site-specific fusion or homologous recombination and have been prevalent in the eastern China for nearly a decade. Comparative genomics showed that CR-hv convergent plasmids, which retain replicons (IncF/IncR) from *bla*_KPC-2_-bearing plasmids, exhibit varying mobility. Plasmids (PC2) primarily composed of the CR environment are conjugative, while those (PC1) rich in hv sequences are non-mobilizable. Although the fitness and spread of these convergent plasmids are not fully understood, their potential circulation in hospital settings raises significant concerns.

In addition to plasmid-borne, *bla*Carb and hv genes were occasionally located on chromosomes in KP via integrated chromosomal elements. This study discovered chromosomal integrations of *bla*_KPC-2_ and hv genes in ST11 strains and revealed the occurrence of transposition events mediated by IS*26*, IS*903* and IS*Esc36*. Yang et al. reported a mobile virulence-encoding fragment, containing *rmpA2*, *iutA* and *iucABCD*, located in the chromosome of a clinical hvKP strain, and identified a transposition event mediated by IS*26* (Yang et al., 2020). Another study from Wang et al. reported the emergence of a clinical *Escherichia coli* ST131 strain carrying a chromosomal *bla*_KPC-2_ gene probably horizontally transferred from the plasmid to the *E. coli* chromosome by the IS*26* element (Wang et al., 2020). Furthermore, our results showed that chromosomes with hv genes are more common than those with *bla*Carbs, hinting at varying fitness costs. The transfer of these genes from plasmids to chromosomes could be driven by the need to reduce the fitness costs associated with plasmid-borne genes, which are less codon-optimized for the host. Recent studies by Lopez et al. suggest that chromosomal integration enables bacteria to optimize these genes for greater energy efficiency and long-term stability (López et al., 2019, 2020). Future research should focus on understanding the fitness, virulence, and adaptability consequences of these transposition events, and explore how chromosomal integration affects the evolution of CRhvKP strains in different environments.

Notably, we screened an ST11 CRhvKP strain (XHKPN391) with *bla*_KPC-2_ and hv genes integrated into the chromosome. This strain was first reported by Zhu et al., who found that the virulence of XHKPN391 was significantly higher than that of other CRhvKP strains carrying only hv plasmids, potentially due to the stable expression of virulence genes integrated into its chromosome (Zhu et al., 2022). This phenomenon may imply heightened complexity in CRhvKP evolution, as chromosomal integration could enhance gene stability, diminish plasmid-dependent transmission, and promote bacterial survival across environments, potentially leading to persistent transmission in healthcare settings (Shawa et al., 2021). However, this strain remains an isolated case and has not reappeared in later strains from the same clonal cluster. Moreover, the integration of the *bla*Carb gene into the chromosome is rarer than that of the hv genes, suggesting that the chromosomal integration of both genes may incur significant adaptive costs and selective pressures.

This study found class 1 integrons in 85% of Chinese CRhvKP strains, predominantly in the form of complete integrons, which carried 14 AMR gene families conferring resistance to various antibiotics, such as aminoglycosides, rifamycins, and beta-lactams including the class B carbapenemases *bla*_IMP_ and *bla*_VIM_, highlighting their role in the spread of MDR and CR (Akrami et al., 2019; Fadare et al., 2023). Significant differences in integron content were observed among major ST types, with over 75% prevalence in ST11 and ST15 isolates, compared to only 45% in ST23, consistent with previous findings by Li et al. on integron variability among KP lineages (Li et al., 2013). ST11, ST15, and ST65 strains predominantly carried complete integrons, known for their capacity to acquire and disseminate AMR genes, while ST23 and ST268 were characterized by In0 and CALIN elements (Néron et al., 2022). Additionally, differences in the integron-related AMR gene profiles across major circulating STs, especially the ST11 and ST15, reflect distinct evolutionary pressures and adaptation strategies among these lineages. These findings underscore the role of integrons in shaping the genomic architecture and resistance profiles of KP, especially in high-risk clones like ST11 and ST15, with the lower prevalence in ST23 possibly explaining its reduced resistance burden.

In conclusion, this study provides a comprehensive genomic analysis of CRhvKP in China, revealing a complex epidemiological and genetic landscape. The diversity of CRhvKP in terms of CR determinants and genetic backgrounds poses a significant challenge to the control of CRhvKP infections. The identification of convergent plasmids carrying both CR and hv traits, along with a chromosomal integration event of these genes, indicates a dynamic evolution of CRhvKP with implications for treatment and infection control. The widespread dissemination of CRhvKP, particularly in eastern China, underscores the urgency for enhanced regional surveillance and intervention strategies. The findings emphasize the need for novel therapeutic strategies, strict infection control measures, and antibiotic stewardship to mitigate the impact of these highly virulent and resistant pathogens, contributing to the global effort to combat antimicrobial resistance.

## Data Availability

The original contributions presented in the study are included in the article/**Supplementary Material**. Further inquiries can be directed to the corresponding authors.

## Supplementary material

Supplementary Figure 1 Frequency distribution of different carbapenemases detected in CRhvKP by sampling year.

Supplementary Figure 2 Frequency proportion of the presence and absence of the *iroBCDN* gene cluster among CRhvKP strains of the five major circulating STs.

Supplementary Figure 3 Boxplot of the number of plasmids carried by each strain within the five major circulating STs of CRhvKP.

Supplementary Table 1 Metadata and genotypes of 1,538 CRhvKP genomes in China.

Supplementary Table 2 cgMLST-based clonal clustering results.

Supplementary Table 3 Metadata of the CR and hv plasmid.
