# Supplementary figures and images for: Molecular epidemiology and genetic dynamics of carbapenem-resistant hypervirulent Klebsiella pneumoniae in China
Source: Front Cell Infect Microbiol. 2025 Feb 14;15:1529929. doi: 10.3389/fcimb.2025.1529929 (PMC11868059; doi:10.3389/fcimb.2025.1529929)

## Carbapenemase

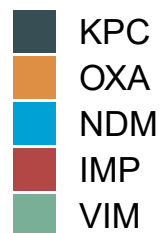

Number of isolates

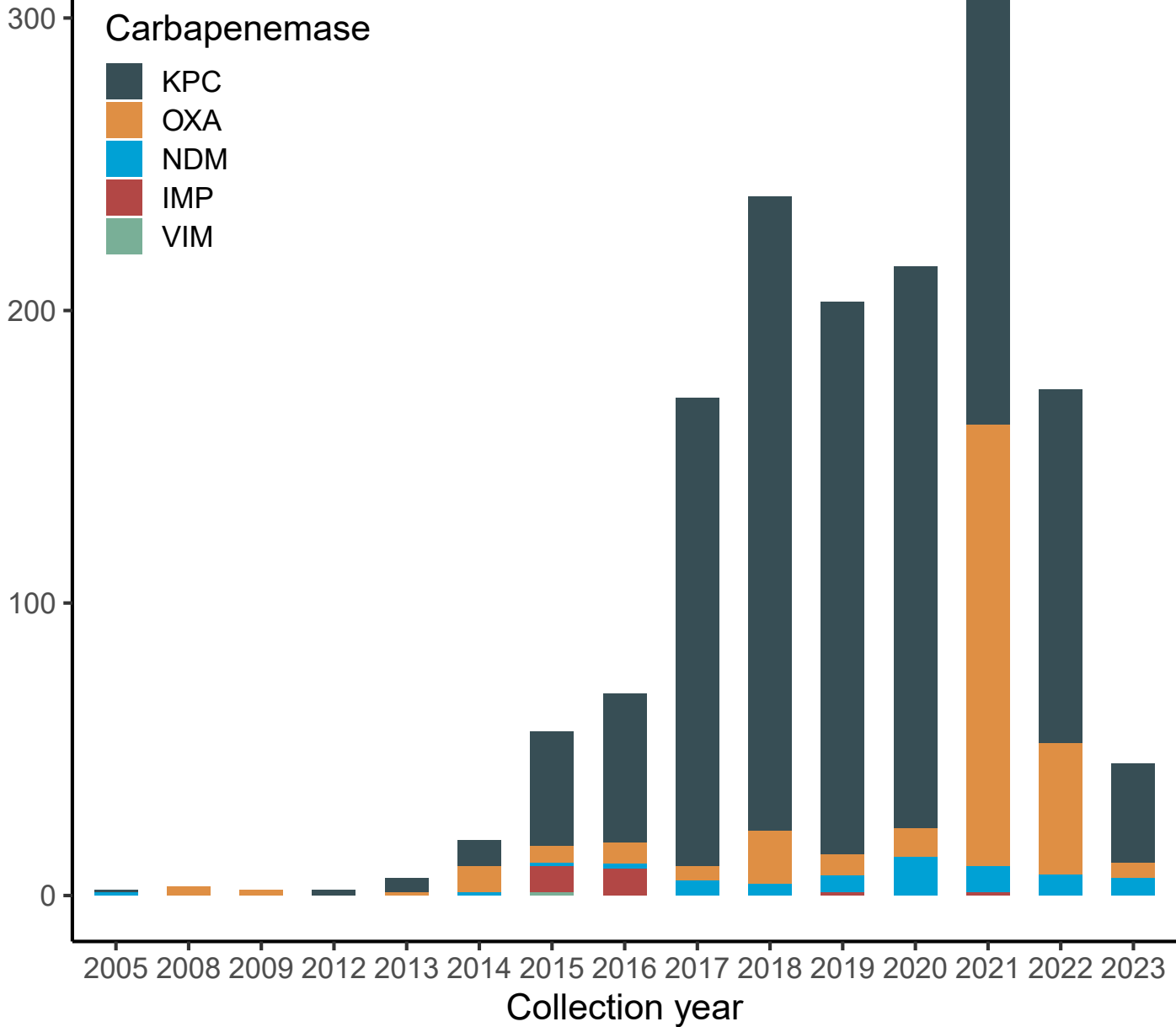

Supplement: Supplementary Figure 1 — Frequency distribution of different carbapenemases detected in CRhvKP by sampling year. [file Image1.pdf]

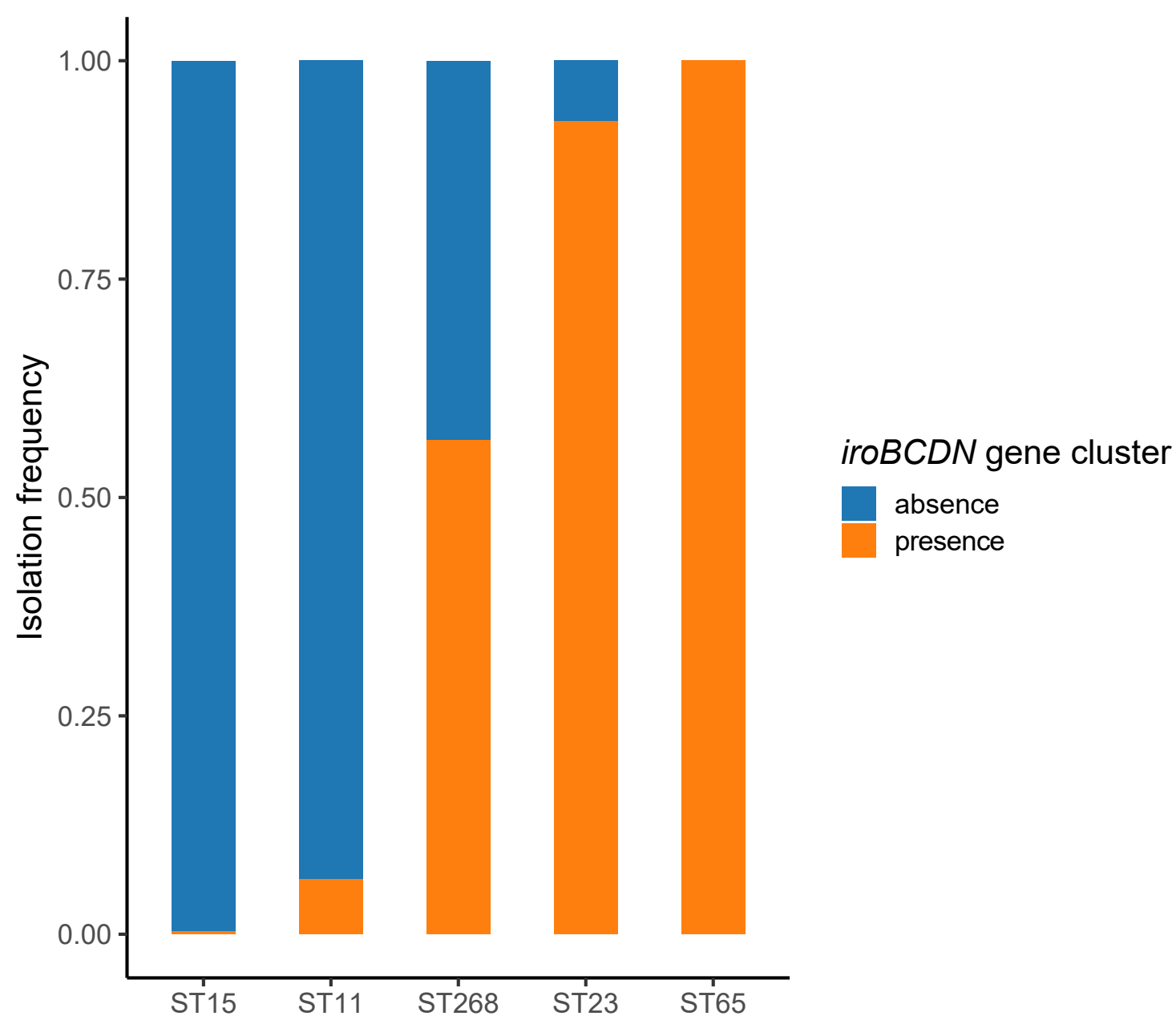

Supplement: Supplementary Figure 2 — Frequency proportion of the presence and absence of the iroBCDN gene cluster among CRhvKP strains of the five major circulating STs. [file Image2.pdf]
