# Supplementary material for: Molecular epidemiology and genetic dynamics of carbapenem-resistant hypervirulent Klebsiella pneumoniae in China
Source: Front Cell Infect Microbiol. 2025 Feb 14;15:1529929. doi: 10.3389/fcimb.2025.1529929 (PMC11868059; doi:10.3389/fcimb.2025.1529929)
Supplement: Supplementary Figure 3 — Boxplot of the number of plasmids carried by each strain within the five major circulating STs of CRhvKP. [file Image3.pdf]

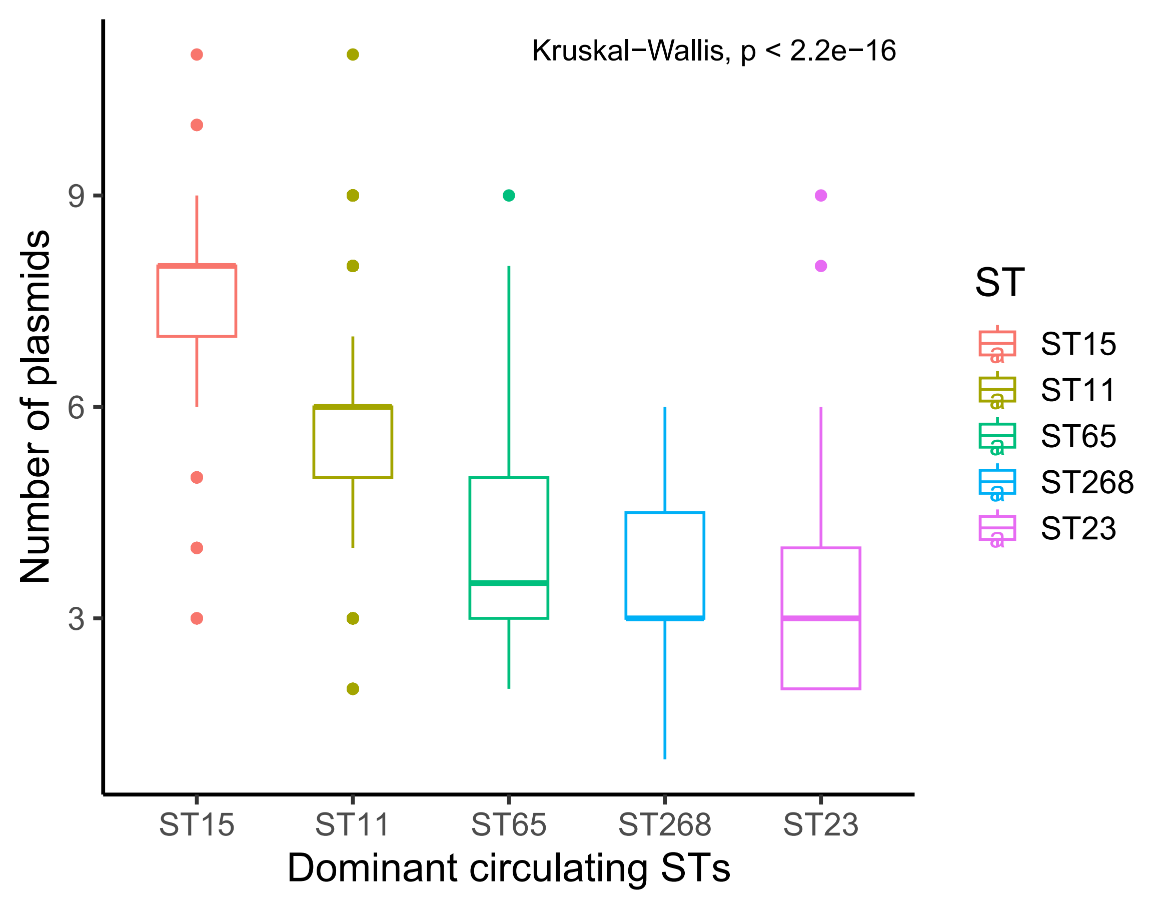
Kruskal−Wallis, p < 2.2e−16
Number of plasmids
9
6
3
ST15
ST11
ST65
ST268
ST23
Dominant circulating STs
ST
ST15
ST11
ST65
ST268
ST23
